# Identification of Proteins Required for Precise Positioning of Apc2 in Dendrites

**DOI:** 10.1534/g3.118.200205

**Published:** 2018-03-30

**Authors:** Alexis T. Weiner, Dylan Y. Seebold, Nick L. Michael, Michelle Guignet, Chengye Feng, Brandon Follick, Brandon A. Yusko, Nathan P. Wasilko, Pedro Torres-Gutierrez, Melissa M. Rolls

**Affiliations:** The Department of Biochemistry and Molecular Biology and The Huck Institutes of the Life Sciences, The Pennsylvania State University, University Park, PA 16802

**Keywords:** dendrite, *Drosophila*, protein localization

## Abstract

In *Drosophila* neurons, uniform minus-end-out polarity in dendrites is maintained in part by kinesin-2-mediated steering of growing microtubules at branch points. Apc links the kinesin motor to growing microtubule plus ends and Apc2 recruits Apc to branch points where it functions. Because Apc2 acts to concentrate other steering proteins to branch points, we wished to understand how Apc2 is targeted. From an initial broad candidate RNAi screen, we found Miro (a mitochondrial transport protein), Ank2, Axin, spastin and Rac1 were required to position Apc2-GFP at dendrite branch points. YFP-Ank2-L8, Axin-GFP and mitochondria also localized to branch points suggesting the screen identified relevant proteins. By performing secondary screens, we found that energy production by mitochondria was key for Apc2-GFP positioning and spastin acted upstream of mitochondria. Ank2 seems to act independently from other players, except its membrane partner, Neuroglian (Nrg). Rac1 likely acts through Arp2/3 to generate branched actin to help recruit Apc2-GFP. Axin can function in a variety of wnt signaling pathways, one of which includes heterotrimeric G proteins and Frizzleds. Knockdown of Gαs, Gαo, Fz and Fz2, reduced targeting of Apc2 and Axin to branch points. Overall our data suggest that mitochondrial energy production, Nrg/Ank2, branched actin generated by Arp2/3 and Fz/G proteins/Axin function as four modules that control localization of the microtubule regulator Apc2 to its site of action in dendrite branch points.

Many differentiated cells have special arrangements of microtubules that are quite different from the most familiar centrosomal organization found in rapidly dividing cells (bartolini and gundersen 2006; muroyama and lechler 2017; sanchez and feldman 2017). Drosophila neurons are a particularly extreme example, and also a good model system for understanding how non-centrosomal microtubule arrays are established and maintained (rolls 2011). Like axons in other animals, those in Drosophila have all plus-end-out microtubules (baas and lin 2011). In stark contrast to the axon, dendritic microtubules are more than 90% minus-end-out (rolls *et al.* 2007; stone *et al.* 2008). This arrangement, with minus ends away from the center of the cell, is very different from “standard” centrosomal microtubule organization in which minus ends are clustered centrally, and thus is particularly intriguing to investigate.

Several different mechanisms that contribute to maintenance of the minus-end-out microtubule array in Drosophila dendrites have been identified. First, local microtubule nucleation in dendrites is important for maintaining neuronal structure (ori-mckenney *et al.* 2012) and uniform polarity (nguyen *et al.* 2014). Second, directed growth of microtubules, or steering, at branch points prevents disruption of polarity by microtubule polymerization (mattie *et al.* 2010). This steering mechanism is the focus here.

Microtubule steering helps maintain minus-end-out polarity in branched dendrites by preventing a microtubule that grows into the branch point from turning away from the cell body at this junction and generating a plus-end-out microtubule (mattie *et al.* 2010; weiner *et al.* 2016). Kinesin-2, a heterotrimeric motor consisting of the motor subunits Klp64D and Klp68D and accessory subunit Kap3, is linked to growing microtubules through binding of Kap3 to the +TIP protein Adenomatous polyposis coli (Apc), which in turn binds the core +TIP EB1 (mattie *et al.* 2010). Kinesin-2 is then positioned on the microtubule plus end with its motor domain capable of engaging with stable microtubules (Figure 1A). If this happens, the plus end can be guided along the stable microtubule to maintain polarity at branch points (weiner *et al.* 2016). Microtubule steering has been reconstituted *in vitro* by linking kinesin motors directly to +TIPs under conditions that allow microtubule polymerization (chen *et al.* 2014; doodhi *et al.* 2014).

**Figure 1:**
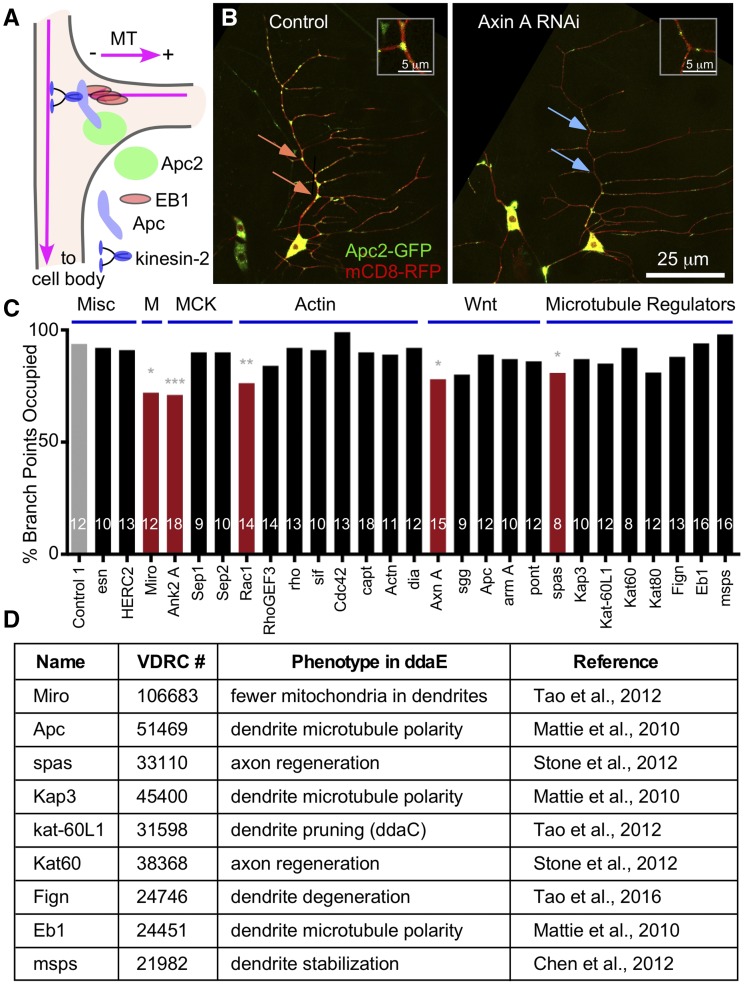
Several proteins are required to position Apc2-GFP at dendrite branch points. (A) A schematic of the microtubule steering mechanism is shown. (B) Images of the ddaE neurons expressing mCD8-RFP and Apc2-GFP are shown for Rtnl2 RNAi (Control 1) (VDRC 33320) and Axin RNAi (VDRC 7748). Orange arrows indicate branch points with high Apc2 signal and blue arrows indicate branch points with low/no Apc2 signal. Insets show the top branch point indicated with an arrow in each panel. (C) Quantification Apc2-GFP branch point occupancy is shown for different RNAi conditions. Titles above the graph indicate which functional groups the RNAi lines belong and are abbreviated as Misc for miscellaneous, M for mitochondria, MCK for membrane cytoskeleton, Actin for actin regulators, Wnt for wnt signaling pathway members, and Microtubule Regulators. The gray bar indicates the control and red bars indicate genotypes that had significantly reduced Apc2 at branch points. Numbers of neurons analyzed are shown within the bars. A Fisher’s Exact test was used to compare each genotype to the control. * *P* < 0.05, ** *P* < 0.01, *** *P* < 0.001. (D) Table of RNAi lines previously shown to have phenotypes in ddaE neurons; the only exception is kat-60L1 which was tested in ddaC not ddaE. The stock number, phenotype, and published reference are noted.

One aspect of the microtubule steering pathway that is not well understood is how the proteins that mediate it are localized to their sites of function at dendrite branch points. One clue is the interaction of Apc with Apc2. Drosophila Apc binds both kinesin-2 and EB1 and so can act as a bridge between the motor and plus end, but it also binds Apc2 (mattie *et al.* 2010). When tagged with GFP, Apc2 localizes strongly to dendrite branch points and can recruit Apc (mattie *et al.* 2010). None of the other proteins in the complex seems to be able to localize to branch points when overexpressed alone. The ability of Apc2 to self-associate (kunttas-tatli *et al.* 2014) may help it to act as a platform to recruit other proteins. Understanding how Apc2 is localized to branch points is thus central to determining how the steering complex (Figure 1) is concentrated where it functions.

## Methods

### Drosophila Stocks and Expression System

Drosophila stocks were obtained in large part from either Vienna Drosophila Resource Center (VDRC) or Bloomington Drosophila Stock Center (BDSC). RNAi lines from the BDSC are part of the TRiP collection; we thank the TRiP at Harvard Medical School (NIH/NIGMS R01-GM084947) for providing transgenic RNAi fly stocks used in this study. Specific RNAi lines, as well as overexpression and mutant alleles, are detailed in Supplemental material, Table S1. The 221-Gal4 driver was used to express transgenes in Class I dendritic arborization sensory neurons. UAS-Dicer2 was included in all RNAi experiments. For whole brain imaging experiments, expression was pan-neuronally driven with elav-Gal4. Stocks with mutant fz alleles including *fz^F31^* and *fz^R52^* were a gift from Dr. Paul Adler at the University of Virginia. Constitutively active UAS-Gαs-GTP and inactive UAS-Gαo-GDP were provided by Dr. Andrew Tomlinson at Columbia University Medical Center. UAS-YFP-Ank2L8 was given to us by Dr. Jan Pielage (Technische Universitat Kaiserslautern). UAS-sggS9A and UAS-sggS9E were obtained from the BDSC. Tester lines for screens included: 1) UAS-dicer2, mCD8-RFP; 221-Gal4, Apc2-GFP, 2) UAS-dicer2, UAS-mCD8-RFP; 221-Gal4, Mito-GFP, 3) UAS-dicer2, UAS-mCD8-RFP; 221-Gal4, UAS-YFP-Ank2L8, 4) UAS-dicer2, UAS-mCD8-RFP; 221-Gal4, UAS-Axin-GFP. Components from each of these lines can be obtained from BDSC. Additional fly lines used were UAS-Arp3-GFP and elav-Gal4, also available at BDSC.

### Confocal Fluorescent in Vivo Microscopy

After mating virgin female flies from tester lines (see *Drosophila* Stocks) with RNAi male flies (crosses kept at 25°), embryos were collected on caps filled with standard media every 24 hr. Caps were incubated with embryos/larvae for 3 days at 25° and used to harvest third instar animals for imaging on the third day. Individual larvae were placed on a microscope slide with a circular piece of dried agar in the middle with a little bit of water. Animals were allowed to move until they were dorsal side up, and then a cover slip was taped down on top of them. 10x objectives were used to locate larvae under the microscope. 60x Oil (NA 1.42) (Olympus) and 63x Oil (NA 1.4) (Zeiss) objectives were used to locate dendritic arborization neurons in the central hemisegments on either side of the animal. For UAS-Apc2-GFP localization, larvae were imaged on an Olympus Fluoview 1000. For the rest of the fluorescent markers including UAS-Mito-GFP, UAS-YFP-Ank2L8, UAS-Arp3-GFP, and UAS-Axin-GFP larvae were imaged on an Olympus Fluoview 1000 or a Zeiss LSM800 scanning confocal microscope.

### Fluorescence Quantification Methods

Images were prepared and quantified using the image processing software Fiji. Maximum projection stacked images were used for quantitation of markers at branch points. UAS-Apc2-GFP and UAS-Mito-GFP experiments were scored with a qualitative binary method. For examples of branch points that were scored as “positive” see branch points indicated with orange arrows throughout the figures, and for those scored as “negative” see examples with blue arrows. For the rest of the markers a relative pixel intensity measurement was used to calculate mean branch point intensity and non-branch point intensity within the main trunk of the comb dendrite. These non-branch point values were then subtracted from branch point to determine the branch point intensity over background. For each marker a set of microscope conditions (laser power, gain, pinhole) was chosen and the same setting were used throughout. The values on the y axes are fluorescence intensity with these settings. For some experiments two y axes are present, and the graph is divided by a dotted line to indicate where the left and right axes apply. The data on each side of the line was collected with a different microscope. Typically the left side is with an Olympus FV1000 and the right side with a Zeiss LSM800. It was necessary to change microscopes in the middle of some of the data sets because the Olympus was destroyed by a flood.

### Statistical Methods

A Fisher’s Exact test to compare each condition to the control was used for UAS-Apc2-GFP screens. Linear or logistic regressions were performed for all other experiments using GraphPad Prism 6 software. Logistic regressions were used because they are the standard for testing differences between probabilities like Apc2-GFP occupancy. Three different control data sets were generated for Apc2-GFP and agree very closely with one another (Figure 1C, 3B and 5C). For many of the graphs the control data from 3B was used. Linear regressions are appropriate for comparing a group to the same control, and so these were used for continuous data sets. Statistical tests were chosen with help from Haley Brittingham as part of her Masters work in Statistics at Penn State. See individual figure legends for statistical test used. Statistical significance is noted as * *P* < 0.05, ** *P* < 0.01, *** *P* < 0.001. All error bars show the standard deviation as this is an intuitive representation of variability. Where no error bars are present the data are categorical.

### Data Availability

Drosophila strains are available upon request. Table S1 contains a list of all fly lines used, and lines with multiple transgenes are listed in the materials and methods. Example raw image files are also available on request.

## Results

### Identification of proteins that localize Apc2 to Dendrite Branch Points

Apc2-GFP localizes robustly to dendrite branch points of Drosophila sensory neurons and can recruit Apc-RFP (mattie *et al.* 2010). In addition, Apc proteins in general act as scaffolds in wnt signaling pathways and so have many known interacting partners (McCartney and Näthke 2008; nelson and nathke 2013). We therefore used Apc2-GFP localization as the readout to identify proteins involved in patterning microtubule regulators within dendrites (Figure 1). Candidates selected to screen included proteins known to interact with Drosophila Apc2, like the formin diaphanous (dia) (webb *et al.* 2009), proteins known to work with Apc in wnt signaling including sgg (GSK3β) (cadigan and peifer 2009), cytoskeletal regulators to reflect the interactions of Apc proteins with both actin and microtubules (dikovskaya *et al.* 2001), and mitochondria as there is evidence Apc can be targeted to them (brocardo *et al.* 2008).

The ddaE sensory neuron was chosen as a model system because it has a simple, stereotyped dendrite arbor (grueber *et al.* 2002); has similar microtubule organization to Drosophila interneurons and motor neurons (stone *et al.* 2008), and previous work on microtubule steering has been done in this cell type (mattie *et al.* 2010). To perform the candidate screen, females from a tester line containing UAS-Dicer2 (to promote neuronal RNAi (dietzl *et al.* 2007)), UAS-mCD8-RFP (to outline the cell), UAS-Apc2-GFP and 221-Gal4 (to drive transgene expression in the ddaE neuron) were crossed to RNAi transgenes. Many Drosophila RNAi lines (including GD and KK lines from VDRC and val1 and val10 lines from the TRiP collection at BDSC) generate RNA hairpins several hundred nucleotides long when transcribed and in the nervous system are typically supplemented with UAS-dicer2 as this enzyme seems limiting in neurons (dietzl *et al.* 2007; ni *et al.* 2009). In contrast, the val20 lines in the TRiP collection generate shRNAs (ni *et al.* 2011) and so can be used without dicer2 in theory, however we tend to see more consistent phenotypes when dicer2 is included, so UAS-dicer2 was included in all RNAi experiments. Larval progeny were mounted on microscope slides and confocal images of ddaE neurons were acquired; one neuron was imaged per animal and approximately 10 neurons were imaged for each RNAi condition. Each branch point along the main trunk of the dorsal comb dendrite was scored as occupied by a bright Apc2-GFP patch or not occupied. All branch points from at least 10 individual neurons were pooled to generate a percent occupancy score. When control RNAi hairpins were expressed, about 90% of branch points were scored as occupied (Figure 1B). Knockdown of Miro, spastin, Rac1, Axin and Ankyrin 2 (Ank2) significantly reduced the percentage of occupied branch points (Figure 1C). While screening we noticed that some of the genotypes resulted in ectopic Apc2-GFP localization, but as our goal was to identify branch point targeting mechanisms, we only scored Apc2 at branch points.

Based on the screen, we selected pathways for additional investigation. Miro links mitochondria to microtubule motors (guo *et al.* 2005) and RNAi targeting Miro reduces the number of mitochondria in ddaE dendrites (tao and rolls 2011), so the reduction of branch point Apc2 in Miro RNAi neurons suggested mitochondria might be involved in Apc2 targeting. The reduction of Apc2 at branch points by Ank2 RNAi suggested the submembrane cytoskeleton might be involved. Of actin regulators tested, only Rac1 RNAi had a phenotype suggesting a specific type of actin arrangement could help recruit Apc2 to branch points. Similarly only one of the wnt pathway proteins tested, Axin, reduced Apc2 at branch points perhaps indicating only one part of the pathway, or a pathway variant, is involved. Of the microtubule regulators tested only spastin (spas) RNAi reduced Apc2 localization. While negative RNAi results are difficult to interpret without detailed analysis of protein levels or additional phenotypes, we have previously found phenotypes in ddaE neurons with some of the same RNAi lines used in this screen. For example, msps RNAi eliminates EB1-GFP comets in ddaE neurons (stone *et al.* 2010) and Kap3 RNAi causes mixed polarity in ddaE dendrites (mattie *et al.* 2010). In fact, most of the RNAi lines used in this screen that target microtubule regulators have described phenotypes in these cells (Figure 1D and (tao and rolls 2011; stone *et al.* 2012; tao *et al.* 2016)). We therefore think that Apc2-GFP can still be localized to dendrite branch points under conditions where microtubules are partially disrupted and focused initially on the other regulators. Interestingly kinesin-2, of which Kap3 is a subunit, has previously been placed upstream of Apc targeting in mammalian axons (ruane *et al.* 2016), but is not upstream in this context.

### Tagged Axin, Ank2 and mitochondria localize to branch points

**Figure 2:**
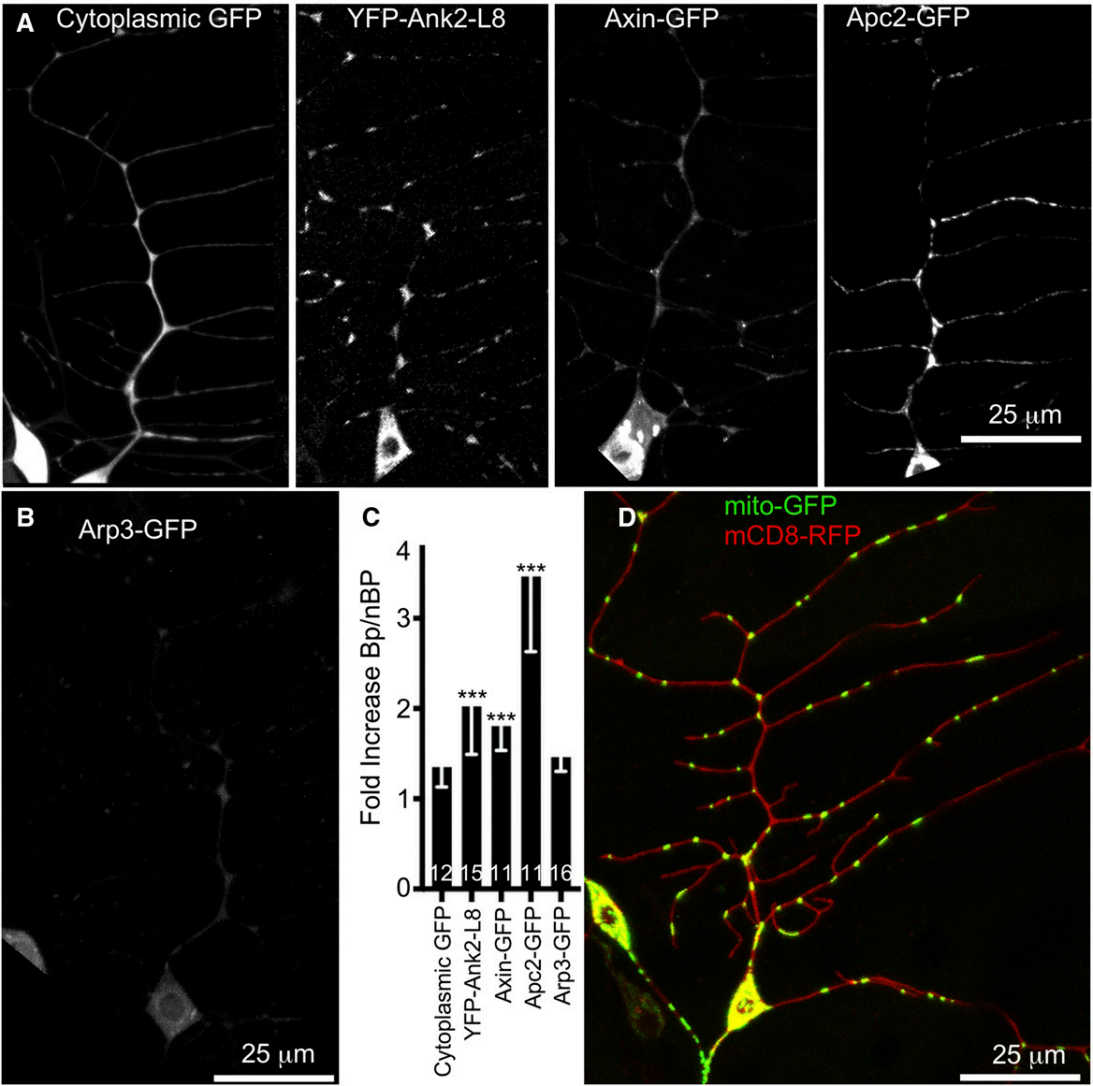
Localization of markers to branch points compared to a non-localized soluble control. (A and B) Representative single channel images of markers used in localization experiments are shown. For comparison a cytoplasmic GFP that fills the dendrite is included. (C) Quantification of fold increase (to allow comparison across markers the branch point average was divided by non-branch point rather than subtracted) in localization between branch point and non-branch point areas of fluorescent markers. Compared to cytoplasmic GFP all except Arp3-GFP are enriched at branch points. Error bars indicate standard deviation. A linear regression was used to determine statistical significance. * *P* < 0.05, ** *P* < 0.01, *** *P* < 0.001. (D) Representative image of mito-GFP distribution in a ddaE neuron.

**Figure 3:**
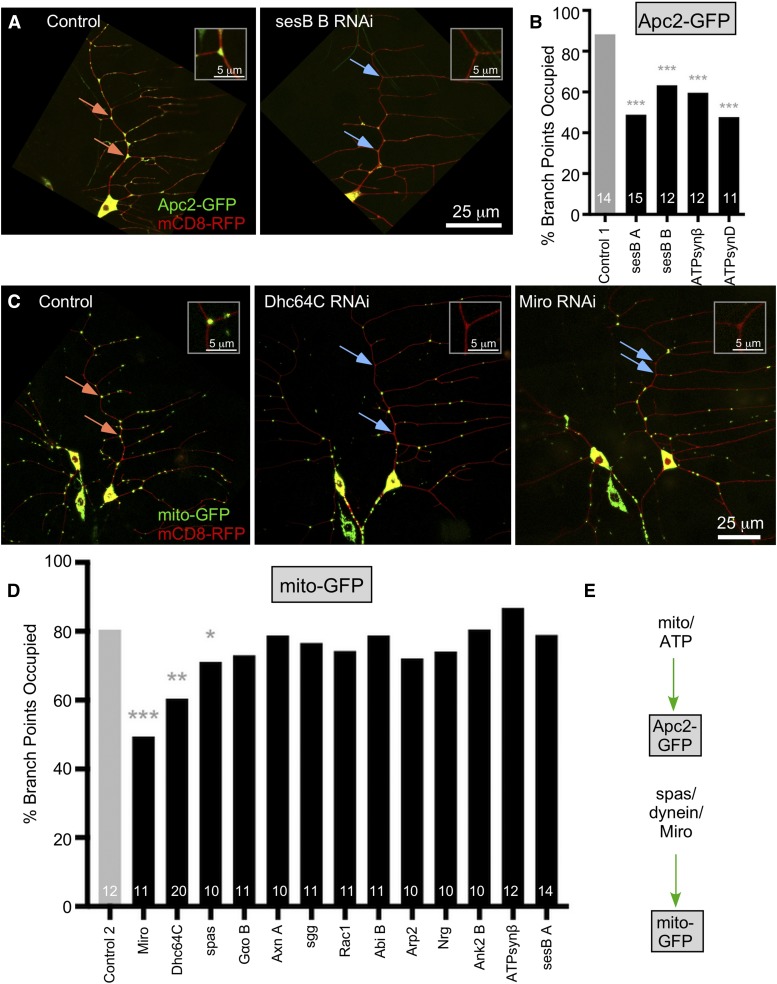
Mitochondrial energy production contributes to Apc2-GFP positioning at branch points. (A) Example images of ddaE neurons expressing Apc2-GFP and mCD8-RFP are shown for control 1 RNAi (VDRC 33320) and sesB RNAi (BL36661). Orange arrows indicate high Apc2 signal and blue indicate low signal. Insets are the top ones indicated with arrows. (B) Quantification of branch point occupancy of Apc2-GFP with RNAi knockdowns. Numbers of neurons analyzed are shown within the bars. A Fisher’s Exact test was used to assess whether conditions were different from the control. (C) Example images of ddaE neurons expressing mito-GFP and mCD8-RFP are shown with Control 1 RNAi (VDRC 33320), Dhc64C RNAi (VDRC 28054), and Miro RNAi (VDRC 106683). Example branch points that contain one or more mitochondria are indicated with orange arrows, and ones without mitochondria are shown with blue arrows. (D) The percentage of branch points occupied by mitochondria along the main comb dendrite is shown in the graph. A logistic regression was used to identify conditions likely to be different from the control. * *P* < 0.05, ** *P* < 0.01, *** *P* < 0.001. (E) A summary diagram of data in the figure is shown.

To begin to determine whether the initial candidate screen identified important regulators of Apc2 dendrite localization, we tested whether any of the positive proteins themselves localized to branch points. To quantitatively assess concentration at branch points, regions of interest between branch points were manually outlined as were those within branch points. The ratio of branch point to non-branch point fluorescence was calculated for cytoplasmic GFP. On average cytoplasmic GFP was about 1.2 fold brighter at branch points than non-branch points, likely reflecting the larger cytoplasmic volume at branch points (Figure 2). A similar analysis of the tagged long exon of Ank2, YFP-Ank2-L8 (pielage *et al.* 2008), indicated it was about twofold brighter at dendrite branch points (Figure 2). Similarly, Axin-GFP (cliffe *et al.* 2003) was about 1.8-fold brighter at branch points (Figure 2). We also examined the distribution of mitochondria in dendrites. While they are fairly evenly distributed throughout the dendrite arbor, the majority of branch points do contain one or more GFP-labeled mitochondria (Figure 2D and 3D). Actin-GFP as well as other tagged actin-binding domains and regulatory proteins, including Arp3-GFP (Figure 2B) were not convincingly localized to branch points. We conclude that a subset of the hits from the initial candidate screen have a localization consistent with functioning to target Apc2 to dendrite branch points.

### Mitochondrial function is required to position Apc2-GFP at branch points

As Miro was required for Apc2 localization and mitochondria localize to most branch points, we further investigated the relationship between mitochondria and Apc2 positioning. We considered two models for the role of mitochondria. In one model, mitochondria might act as a physical docking platform for Apc2, as suggested for some cancer-associated human Apc truncation mutants (brocardo *et al.* 2008). In a second model, mitochondria could function primarily as a local source of ATP. To test whether mitochondrial ATP production might be important in this context, we knocked down two proteins that play roles in oxidative phosphorylation. SesB is an ADP/ATP antiporter that allows exchange of ATP and ADP across the inner membrane of the mitochondrion and ATP synthase beta (ATPsynβ) is a subunit of the complex that generates ATP from ADP. Targeting transcripts that encode either of these proteins by RNAi reduced the occupancy of branch points by Apc2-GFP (Figure 3A and B). This result suggests that energy production by mitochondria is important for Apc2 localization (Figure 3E).

In addition to investigating how mitochondria might be involved in Apc2 positioning, we wished to determine whether any of the other factors we identified in our initial screen might influence Apc2 localization indirectly by acting upstream of mitochondrial positioning. We generated a tester line that contained mito-GFP (UAS-Dicer2, UAS-mCD8-RFP; 221-Gal4, UAS-mito-GFP) and crossed it to RNAi lines that reduced Apc2 localization including lines targeting Axin, Rac1 and Miro. In control ddaE neurons about 80% of branch points along the main backbone of the comb dendrite contained mitochondria (Figure 3C and D). This occupancy is slightly lower than that of Apc2-GFP, consistent with the idea that mitochondria do not act directly as a platform for Apc2. Proteins known to be involved in mitochondrial transport into dendrites reduced dendritic branch point localization of mito-GFP as expected: Miro is required for mitochondrial transport into axons and dendrites (guo *et al.* 2005; tao and rolls 2011; babic *et al.* 2015), and dynein (Dhc64C) is required to transport mitochondria into dendrites in Drosophila (satoh *et al.* 2008). Knockdown of spastin also reduced branch point localization of mitochondria, and the RNAi line used here is one we have previously shown has phenotypes similar to mutants (stone *et al.* 2012). Spastin could influence mitochondrial positioning either through its role in microtubule organization or through its role in ER positioning, as both functions can be important in Drosophila neurons (sherwood *et al.* 2004; rao *et al.* 2016), and mitochondria are closely linked to the ER and microtubules (Labbé *et al.* 2014). We conclude that the effects of Miro and spastin on Apc2-GFP localization are likely due to their role in localization of mitochondria to dendrite branch points. However, other proteins like Axin and Ank2 probably influence Apc2 localization independently from mitochondria.

### Ank2 works With Neuroglian to position Apc2 at branch points

The initial screen suggested a requirement for Ank2 in Apc2-GFP localization to dendrite branch points (Figure 1). Ank2 has been described to function in the axon near the cell body (yamamoto *et al.* 2006; jegla *et al.* 2016) and in more distal axons and terminals (koch *et al.* 2008; pielage *et al.* 2008; stephan *et al.* 2015), but not dendrites. However, the localization of YFP-Ank2-L8 was consistent with a dendritic role (Figure 2A).

**Figure 4:**
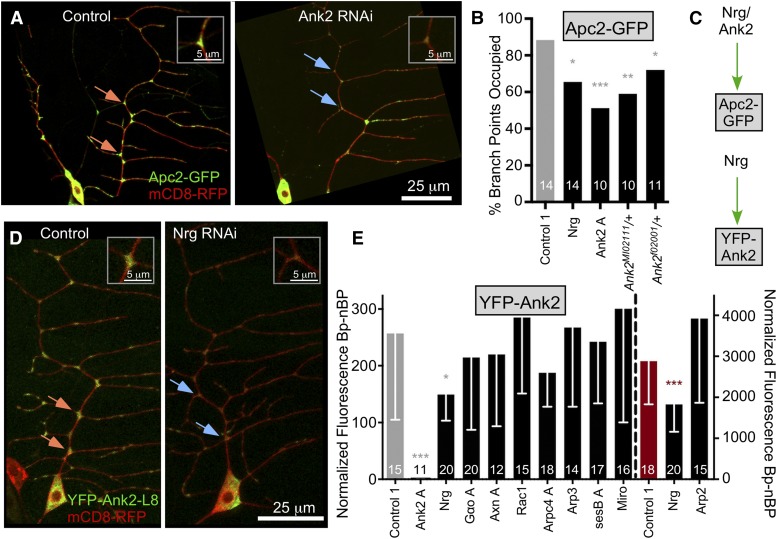
Ank2 and Neuroglian help position Apc2-GFP at branch points. (A) Example images of Apc2-GFP and mCD8-RFP in ddaE neurons with Control1 RNAi (VDRC 33320) or UAS-Ank2 RNAi (VDRC 107369) are shown. (B) Quantification of Apc2-GFP localization is shown in the graph; numbers on the bars are neurons analyzed. The control dataset is the same one used in Figure 3B. A Fisher’s Exact test was used to determine statistical significance. Throughout the figure orange arrows indicate branch points with high fluorescence and blue arrows low fluorescence. Insets show top examples. (C) The diagram summarizes data in the figure. (D) Images depicting UAS-YFP-Ank2L8 distribution in ddaE neurons with either Control 1 RNAi (VDRC 33320) or Nrg RNAi (VDRC 107991). (E) Quantification of YFP-Ank2L8 intensity at branch points compared to non-branch points is shown. The gray bar indicates control cells imaged on an Olympus FluoView 1000 and the red bar indicates the same control genotype imaged on a Zeiss LSM800. All data to the left of the dashed line was collected with the Olympus microscope, while data to the right was collected with the LSM800. The axes to the left and right are for the two different microscopes. Error bars are standard deviation. Comparisons were made between data collected on a single microscope, and the color of the star indicates which control is used for comparison. A linear regression was used to determine statistical significance. * *P* < 0.05, ** *P* < 0.01, *** *P* < 0.001.

To confirm the involvement of Ank2 in Apc2 positioning in dendrites, we took several approaches. First, we retested Ank2 RNAi (Figure 4A and B). Second, we used two different mutant alleles of *Ank2* and crossed these to the Apc2 tester line to generate animals with one normal copy of the *Ank2* gene and one mutant copy; Apc2-GFP was reduced at branch points in both backgrounds (Figure 4B). Finally, we targeted Neuroglian (Nrg) by RNAi. Nrg is a membrane protein partner of Ank2 in other contexts (bouley *et al.* 2000; yamamoto *et al.* 2006). Nrg RNAi also reduced Apc2-GFP branch point occupancy. Thus multiple lines of evidence indicate that Nrg and Ank2 are required to position Apc2 (Figure 4C). Note that neither Nrg RNAi nor one Ank2 RNAi reduced mitochondrial localization to branch points (Figure 3D).

Drosophila Ank2 is expressed primarily in neurons (bouley *et al.* 2000) and contains extremely long exons that generate giant L and XL isoforms (koch *et al.* 2008; pielage *et al.* 2008). These giant Ank2 isoforms have a common evolutionary origin and overlapping function with vertebrate giant ankyrins (jegla *et al.* 2016). The *Ank2^f02001^* allele is a characterized *P* element insertion in the exon that encodes the L region, and it specifically reduces this splice form (koch *et al.* 2008; pielage *et al.* 2008). Similarly the RNAi line labeled Ank2 A targets the L region. In contrast, the *Ank2^MI02111^* transposon insertion disrupts the conserved ankyrin core and so reduces all isoforms (see Table S1 and FlyBase). The fact that one copy of the *Ank2^f02001^* allele and the Ank2 A RNAi reduced Apc2 branch point localization (Figure 4B) suggested that the L form is involved in this Ank2 function. The exon that encodes the region specific to the L form is the one contained in YFP-Ank2-L8.

To determine whether Ank2 was likely to act downstream of any of the other proteins required for Apc2 localization, we generated a tester line with 221-Gal4, mCD8-RFP, YFP-Ank2-L8 and Dicer2 and crossed flies from this line to a variety of RNAi transgenic flies. For all genotypes images were acquired at the same microscope settings (within each data set) and the average intensity between branch points was subtracted from that at branch points (Figure 4D and E). As a control, the Ank2 A RNAi line that targets the coding sequence for the L region was used and it completely eliminated fluorescence of YFP-Ank2-L8 (Figure 4E). Nrg RNAi also reduced YFP-Ank2-L8 signal at branch points (Figures 4D and E) consistent with Nrg and Ank2 working together. None of the other RNAi lines tested reduced the branch point localization of YFP-Ank2-L8 (Figure 4C and D), although in other scenarios G proteins and fz can act through Ank2 (luchtenborg *et al.* 2014). Based on this data, the simplest model is that Nrg helps concentrate Ank2-L at branch points, and Nrg and Ank2-L function to position Apc2 independently of mitochondria and other regulators (Figure 4C).

### Regulators of branched actin are required for Apc2-GFP branch point localization

**Figure 5:**
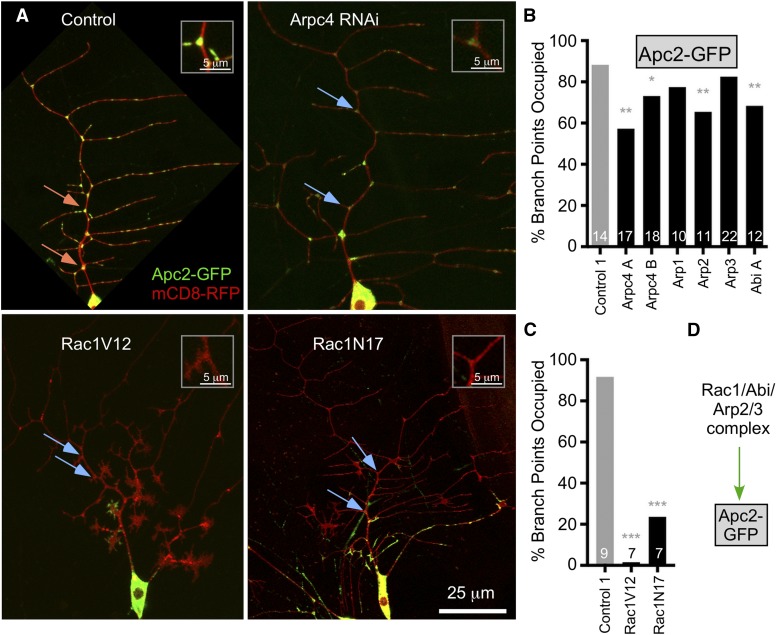
Arp2/3 complex members help recruit Apc2-GFP to branch points. (A) Example images of Apc2-GFP and mCD8-RFP in ddaE neurons with Control 1 (VDRC 33320) RNAi or Arpc4 RNAi (BL41888). See Table S1 for a full listing of all the RNAi line numbers. Lower images show Apc2-GFP with overexpressed Rac1V12 (GTP-bound) and Rac1N17 (GDP-bound). (B) Quantification of Apc2-GFP branch point occupancy with Arp2/3 complex RNAis; the control is the same as in Figure 3B. Numbers on the graph are numbers of cells analyzed and a logistic regression was used to determine significance, * *P* < 0.05, ** *P* < 0.01, *** *P* < 0.001. (C) The Apc2-GFP tester line was crossed to the control 1 RNAi (same genotype as in B, but different set of animals) or UAS-controlled Rac1 mutants. Numbers of animals tested for each condition are shown in the bars, and a logistic regression was used to analyze the data. The p values are indicated as in B. (D) A summary of results in the figure is diagrammed. In all panels orange arrows indicate occupied branch points and blue ones show branch points scored as unoccupied.

Along with Ank2 and mitochondria, our initial Apc2 localization screen indicated that the small GTPase Rac1 helps recruit Apc2 to branch points (Figure 1C). While Rac1 can regulate many different signaling cascades, its classic role is to stimulate generation of branched actin formation by the Arp2/3 complex through activation of the WAVE complex (bosco *et al.* 2009; derivery and gautreau 2010). We therefore tested Arp2/3 and WAVE complex members for a role in Apc2-GFP targeting to branch points. Arp2/3 complex members tested included Arp1, Arpc4, Arp2 and Arp3. The majority of RNAi lines that targeted these proteins reduced Apc2 GFP localization at dendrite branch points (Figure 5A and B). There were several RNAi lines that did not have an effect (Figure 5B), perhaps because they did not knock their targets down as efficiently as some of the others. We tested Abi as a representative of the WAVE complex and it also reduced Apc2-GFP branch point localization (Figure 5B). Thus generation of branched actin by Arp2/3 nucleation seems to be required for Apc2-GFP targeting in dendrites (Figure 5D). To confirm that Rac1 is involved in Apc2-GFP localization, we expressed constitutively GDP-bound Rac1^N17^ and constitutively GTP-bound Rac1^V12^ (luo *et al.* 1994). Both forms of Rac1 dramatically reduced branch point occupancy by Apc2-GFP (Figure 5A and C), but also had strong effects on dendrite architecture (Figure 5A). We conclude that cycling of Rac1 between GTP and GDP bound forms is likely important for Apc2 localization, but that Rac1 also affects the dendritic cytoskeleton more broadly.

Based on this data, the simplest model is that a patch of branched actin is generated at the branch point itself. To see if we could get any direct evidence for this, we expressed tagged actin and Arp2/3 complex members in the ddaE neuron. While all of the markers tested were present at branch points, for most it was not clear if they were more concentrated at branch points than soluble GFP. For example, Arp3-GFP (hudson and cooley 2002) can be seen in dendrites (Figure 2B), but is not significantly enriched at branch points compared to control soluble GFP (Figure 2C). We were therefore not able to screen for players acting upstream of branched actin, or get more direct evidence that branched actin is generated locally to recruit Apc2-GFP.

### A subset of wnt signaling proteins acts through Axin to localize Apc2 to dendrite branch points

**Figure 6:**
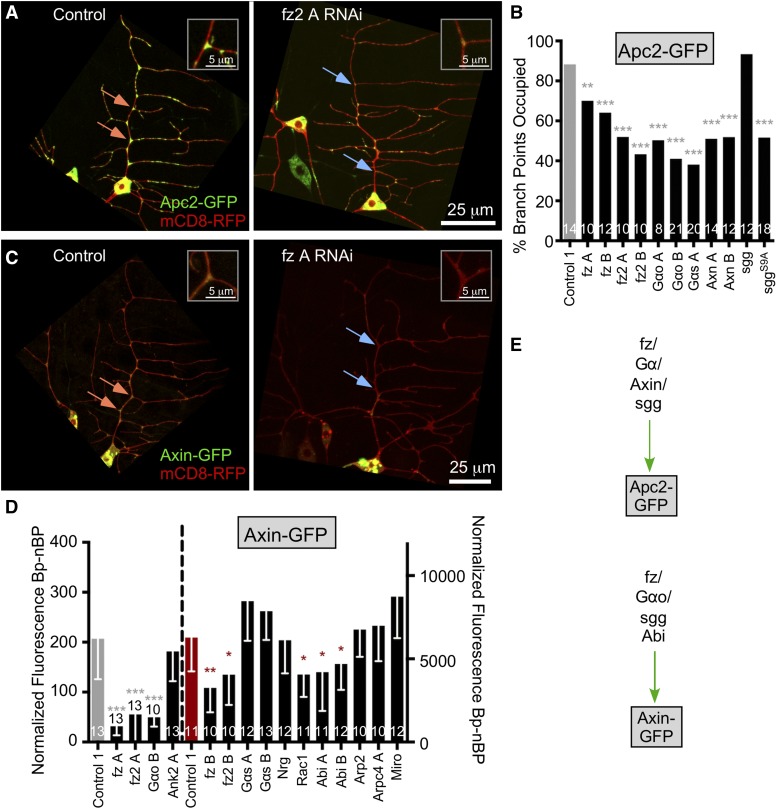
Wnt signaling proteins localize Apc2 and Axin to dendrite branch points. (A) Example images of ddaE neurons expressing Apc2-GFP and mCD8-GFP with Control 1 (VDRC 33320) RNAi or fz A RNAi (VDRC 105493). (B) Quantification of Apc2-GFP branch point occupancy with RNAi knockdown of wnt signaling proteins compared to the same control dataset used in Figure 3B. A Fisher’s Exact test was used to determine significance ** *P* < 0.01, *** *P* < 0.001. (C) Images of Axin-GFP in ddaE neurons with Control 1 (VDRC 33320) RNAi or fz A RNAi (VDRC 105493). (D) Quantification of Axin-GFP intensity at branch points minus intensity between branch points is shown in the graph. Gray and red controls indicate imaging done on an Olympus Fluoview 1000 (gray) or Zeiss LSM800 (red). Dashed line separates two halves of the graph. A linear regression was used to determine statistical significance, * *P* < 0.05, ** *P* < 0.01, *** *P* < 0.001; stars are color-coded to indicate the appropriate control. Error bars are standard deviation. Throughout the image panels orange arrows point out branch points with high fluorescence, and blue ones low fluorescence. (E) A summary of results in the figure is shown.

In our initial screen Axin RNAi reduced localization of Apc2-GFP to dendrite branch points (Figure 1). Axin is a scaffolding protein that plays a central role in wnt signaling (cadigan and peifer 2009; nusse and clevers 2017), so we tested other proteins linked to wnt signaling for a role in Apc2-GFP localization. In the initial screen RNAi lines targeting armadillo (β-catenin) and sgg (GSK3β), key players in canonical wnt signaling, did not have phenotypes (Figure 1). We retested sgg RNAi, and also used a mutant, sggS9A, which eliminates a negative regulatory phosphorylation site and makes the kinase more active (hazelett *et al.* 1998). Again, the RNAi had no phenotype, but overexpression of sggS9A reduced Apc2 branch point localization (Figure 6B) suggesting sgg might at least be able to negatively regulate proteins involved in Apc2 localization in dendrites. Two different RNAi lines targeting each of the wnt receptors frizzled (fz) and frizzled2 (fz2) reduced Apc2-GFP localization (Figure 6B); they also appeared to increase branching of distal dendrites, but did not affect morphology of the main dendrite trunk where quantitation was performed. For fz the two large RNAi hairpins target different regions of the gene, and for fz2 both target the same region although they were generated independently (Figure S1). Although best known for their role regulating β-catenin destruction, frizzleds are 7-transmembrane domain proteins and can function as G-protein coupled receptors (koval *et al.* 2011), transduce fz signals in the wing (katanaev *et al.* 2005) and interact with Axin (egger-adam and katanaev 2010). We therefore tested several G-protein alpha subunits as well. Reduction of Gαo and Gαs by RNAi reduced Apc2-GFP localization to dendrite branch points (Figure 6B). Thus core elements of a wnt signaling pathway variant are involved in Apc2-GFP localization.

To determine which candidates might act upstream of Axin in the Apc2 localization pathway, we generated a tester line that contained UAS-dicer2, UAS-mCD8-RFP; 221-Gal4, UAS-Axin-GFP and crossed this to RNAi transgenic flies. Two different RNAi lines targeting fz and fz2 reduced Axin-GFP localization to branch points (Figure 6D). Targeting Gαo, but not Gαs, also reduced Axin targeting. Thus frizzleds may work through Gαo to regulate Axin in this context as suggested by studies in other Drosophila tissues (egger-adam and katanaev 2010). In contrast, Miro, Ank2 and Nrg RNAi did not reduce Axin localization suggesting that Nrg/Ank2 and local mitochondrial function are not required upstream of Axin. We could not make a conclusion about whether branched actin regulates Axin positioning because of mixed results: RNAis targeting Arp2/3 components did not have a phenotype in this assay, despite being required for Apc2-GFP localization (Figure 5B), however, RNAis targeting Rac1 and Abi (two independent RNAis targeting different gene regions; see Figure S1), upstream regulators of Arp2/3, did affect Axin localization. Both Rac1 and Abi have roles outside Arp2/3 regulation so it is possible they are acting in some other way, or that Axin-GFP localization is slightly more resistant to perturbation by changes in actin than Apc2 localization. A summary diagram of results in the figure is shown in panel 6E.

**Figure 7:**
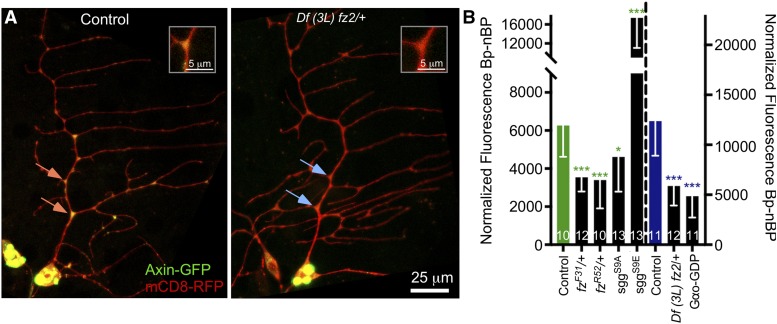
Dominant negative and mutant approaches confirm the role of GαO, fz and sgg in Axin targeting. (A) Example images of ddaE neurons expressing Axin-GFP and mCD8-RFP with Control (yw) or Df (3L) fz2 (BL 6754). Orange arrows point out branch points with high fluorescence, and blue ones low fluorescence. (B). Quantification of Axin-GFP branch point intensity compared to non-branch point intensity is shown. The green bar indicates control taken on an inverted LSM800. The blue bar indicates the same control taken on an upright LSM800; absolute fluorescence values vary based on the particular microscope. A dashed line separates the bars that correspond to the left or right axis respectively. A linear regression was used to determine statistical significance, * *P* < 0.05, ** *P* < 0.01, *** *P* < 0.001 and error bars are standard deviation.

To confirm the involvement of a variant wnt signaling pathway, we used mutant and dominant negative approaches in addition to RNAi. *Df(3L)fz2* is a small deficiency that disrupts the *fz2* and *rept* genes (bhanot *et al.* 1999; ihry and bashirullah 2014). The *fz^R52^* allele has an early stop codon and is a strong loss of function mutant that makes very little protein (jones *et al.* 1996) and *fz^F31^* allele is a point mutation P278S (jones *et al.* 1996) and has a relatively weak phenotype for both canonical and planar cell polarity wnt signaling (povelones *et al.* 2005). Heterozygosity for either *fz* allele or the *fz2* deficiency reduced Axin-GFP localization to branch points (Figure 7). We also used a GDP-bound form of Gαo (katanaev *et al.* 2005) to confirm the involvement of this G protein in Axin localization, and observed a reduction of Axin-GFP at branch points. As for Apc2-GFP, the activated sggS9A reduced Axin-GFP localization (Figure 7), and expression of the constitutively inhibited sggS9E (bourouis 2002) increased Axin-GFP localization again suggesting a negative role for sgg.

Based on the results so far, we have identified four regulatory modules that cooperate to position Apc2-GFP to dendrite branch points: 1) local ATP production by mitochondria, 2) Nrg/Ank2, 3) branched actin, and 4) fz/Gαo/Axin. The data also suggest that these modules likely act independently with the following possible exceptions: 1) we could not determine whether any of the modules act upstream of actin, and 2) we could not exclude that actin acts upstream of Axin.

## Discussion

**Figure 8:**
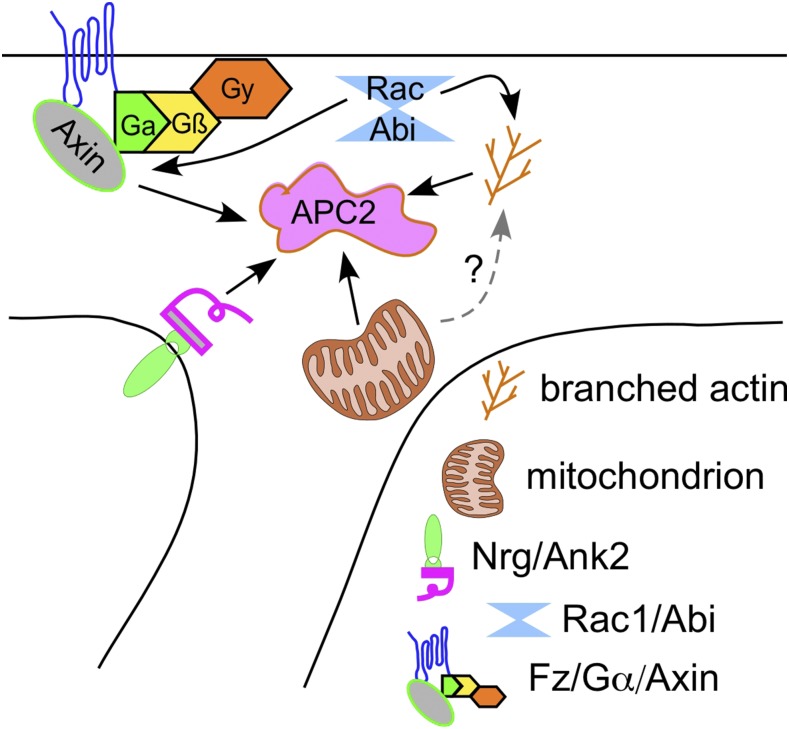
Summary of proteins involved in Apc2-GFP localization to dendrite branch points. The figure is a model that assembles the different proteins/organelles required to position Apc2-GFP at dendrite branch point. The small summary diagrams in figures throughout represent the dependencies that were used to generate this model.

Generation of a minus-end-out microtubule array in Drosophila dendrites involves activity of both plus and minus end regulators at dendrite branch points (mattie *et al.* 2010; nguyen *et al.* 2014). To understand how these regulators are concentrated at branch points, we began with Apc2-GFP as it can recruit Apc and is very robustly targeted (mattie *et al.* 2010). We started by sampling a variety of candidates, from Apc interactors to representatives of different cytoskeletal systems. Positives in this screen spread across different groups of proteins, rather than pinpointing a single regulatory pathway (Figure 1). Tagged versions of several of the proteins that emerged from the initial screen, including Axin and Ank2, also localized to dendrite branch points. After extensive secondary screening to validate the initial screen and order hits into dependency groups, we propose that they can be put into four functional modules, each of which is required for Apc2-GFP concentration at dendrite branch points (Figure 8).

Of these, the one that made the most intuitive sense was Rac1/Abi/Arp2/3. In Drosophila neuroblasts, Apc2 is localized at the cell cortex (akong *et al.* 2002), which tends to be a region rich in actin. Cortical actin is nucleated by Arp2/3 and formins (bovellan *et al.* 2014), though in dendrites we only found a role for Arp2/3 in Apc2 localization. Actin has also been linked more directly to Apc2 localization in several other contexts. During the syncytial divisions of early Drosophila embryos, Apc2 localizes with actin dynamically (mccartney *et al.* 2001). In this case, however, armadillo (β-catenin) was proposed to link actin to Apc2 (mccartney *et al.* 2001) and we do not have evidence for a role for armadillo here. Apc2 also localizes to the cortex of Drosophila S2 cells in culture, but in this case Axin is not thought to play an upstream role (zhou *et al.* 2011) so it is unclear whether the link between Apc2 and actin is mechanistically similar in dendrites and the cortex of other cells. Alternatively, Arp2/3 could influence Apc2 localization through its role in endocytosis (galletta and cooper 2009). This idea is particularly appealing because mammalian Apc can bind the clathrin adaptor AP-2 mu1 subunit (matsui *et al.* 2008). A potential role for endocytosis is also worth considering because ankyrins also interact with endocytic machinery, either directly through binding proteins that regulate endocytosis, or indirectly by helping to organize a submembrane spectrin network that opposes endocytosis (bennett and lorenzo 2016). The neuronal ankyrin, Ank2, was also a hit in our initial screen, and Nrg, a plasma membrane protein partner of Ank2, acts upstream of Ank2. In general ankyrins link membrane proteins to the submembrane spectrin network, which can be regionally specialized as in the axon initial segment (AIS). In the AIS in Drosophila Ank2 and Neuroglian are required to establish a plasma membrane diffusion barrier that helps pattern membrane proteins (jegla *et al.* 2016). So an alternate potential role to regulation of endocytosis for Ank2 and Neuroglian, is making a region of the plasma membrane distinct, as they do at the axon initial segment (jegla *et al.* 2016). However, while we identified the plasma membrane proteins fz and fz2, as well as lipid anchored heterotrimeric G proteins, as regulators of Apc2 localization, we do not think that Ank2 acts by partitioning any of these players within branch points because they act upstream of Axin localization and Ank2 does not.

In addition to the Arp2/3 and Ank2/Nrg modules, mitochondria are important for Apc2 localization. Both actin polymerization activated by Arp2/3 and cycling of heterotrimeric G proteins are potential energy consumers. However, as mitochondria did not affect Axin localization, but heterotrimeric G proteins did, actin is more likely to be the target of ATP production by mitochondria. Alternatively there could be yet another process occurring at the branch point that requires local energy production.

The fourth module acts through Axin to position Apc2. While Axin itself was not a surprise as it binds Apc2 (roberts *et al.* 2011), the involvement of heterotrimeric G proteins and frizzleds upstream of Axin was not expected. First, it was surprising that plasma membrane proteins, Neuroglian, fz and fz2, would be involved in positioning Apc2, a cytosolic protein involved in steering microtubules. Second, it is only quite recently that frizzleds have been accepted to function as GPCRs (koval *et al.* 2011; nichols *et al.* 2013), so the involvement of heterotrimeric G proteins was not a given. Third, although frizzleds, G proteins and Axin have been linked in Drosophila, this work has been done primarily in epithelial cells (katanaev *et al.* 2005; egger-adam and katanaev 2010) and there was no evidence that this pathway also might function in dendrites. However, the data strongly indicates that frizzleds, Gαo and Gαs act to position Apc2, and all except Gαs likely act through Axin as they are required for its positioning. The ability of activated and inactive forms of sgg (GSK3β) to modulate localization of Apc2-GFP and Axin-GFP are consistent with a subset of wnt signaling proteins playing a role in branch point localization as sgg can bind Axin (kremer *et al.* 2010) as well as other wnt signaling proteins. However, we do not have evidence that sgg is normally involved in branch point localization at this point because the RNAi that targets sgg had no effect in any of the assays. Based on the data, this wnt pathway variant seems to have Apc2 localization as its output. The only known dendritic function of Apc2 is microtubule steering. So, in contrast to canonical wnt signaling, which regulates transcription through β-catenin, this pathway seems to act locally to regulate the cytoskeleton.

## Supplementary Material

Supplemental material is available online at www.g3journal.org/lookup/suppl/doi:10.1534/g3.118.200205/-/DC1.
