# Supplementary material for: Identification of Proteins Required for Precise Positioning of Apc2 in Dendrites
Source: G3 (Bethesda). 2018 Mar 30;8(5):1841–53. doi: 10.1534/g3.118.200205 (PMC5940173; doi:10.1534/g3.118.200205)
Supplement: Supplementary file 1 [file 1841FigureS1.pdf]

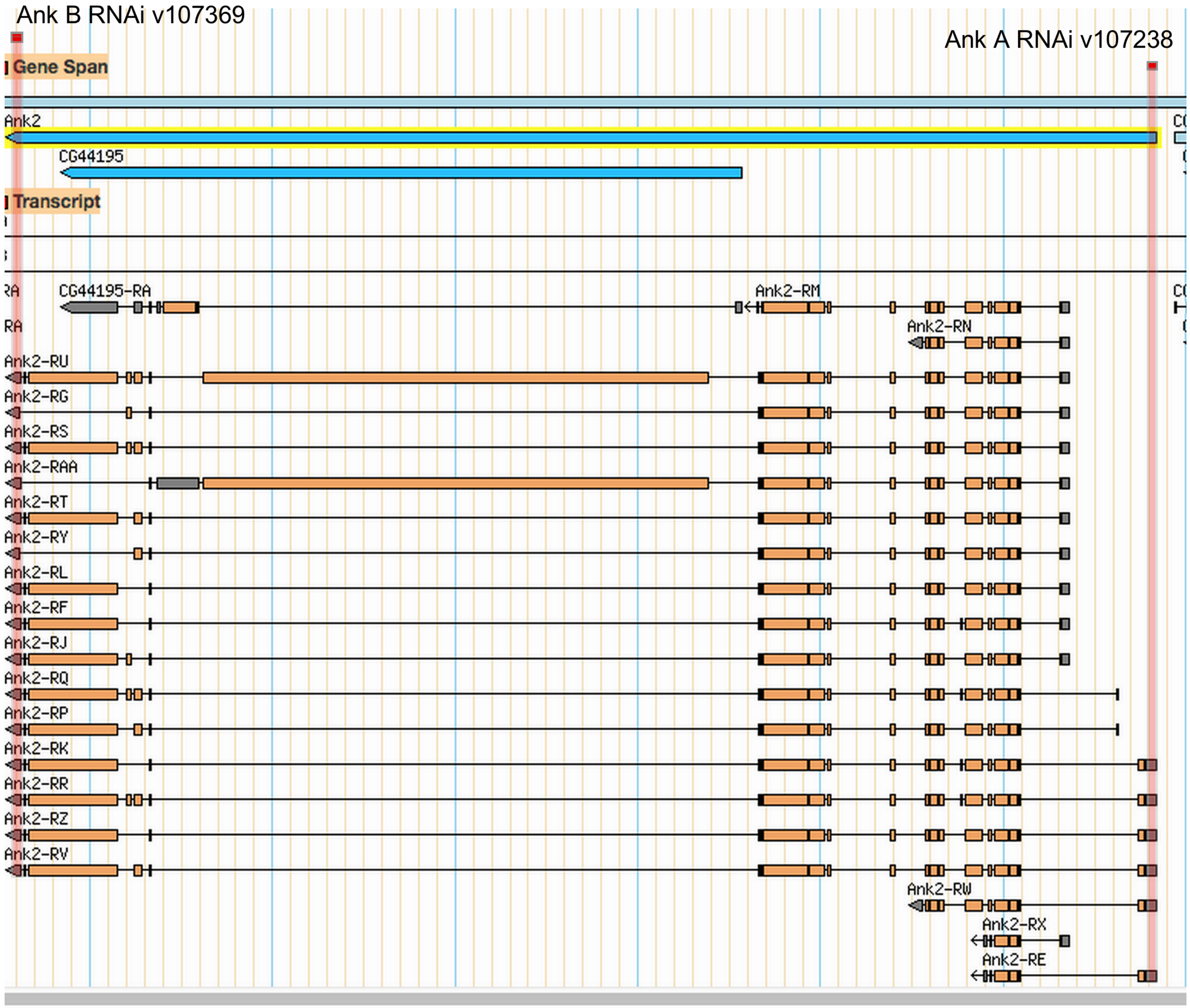
Ank B RNAi v107369
Ank A RNAi v107238
Gene Span
Ank2
CG44195
Transcript
CG44195-RA
Ank2-RM
Ank2-RN
Ank2-RU
Ank2-RG
Ank2-RS
Ank2-RAA
Ank2-RT
Ank2-RY
Ank2-RL
Ank2-RF
Ank2-RJ
Ank2-RQ
Ank2-RP
Ank2-RK
Ank2-RR
Ank2-RZ
Ank2-RV
Ank2-RW
Ank2-RX
Ank2-RE

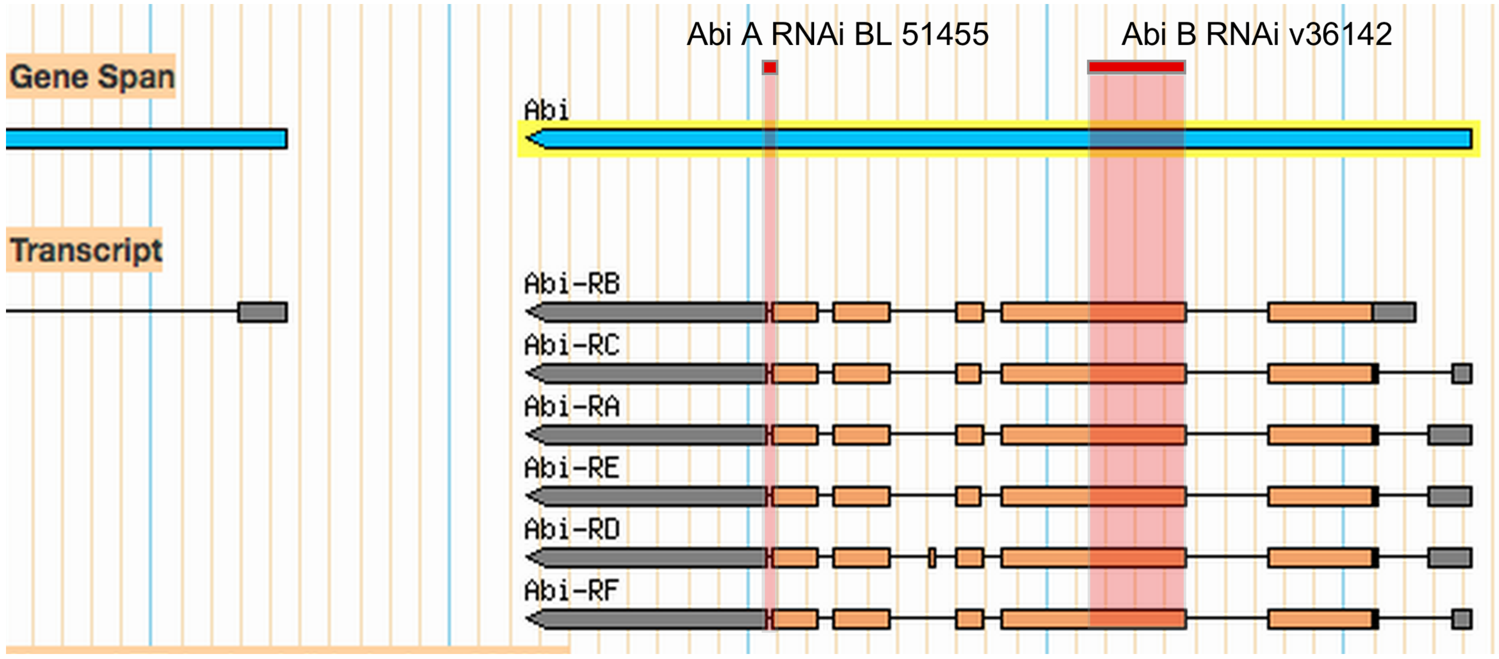
Abi A RNAi BL 51455
Abi B RNAi v36142
Gene Span
Abi
Transcript
Abi-RB
Abi-RC
Abi-RA
Abi-RE
Abi-RD
Abi-RF

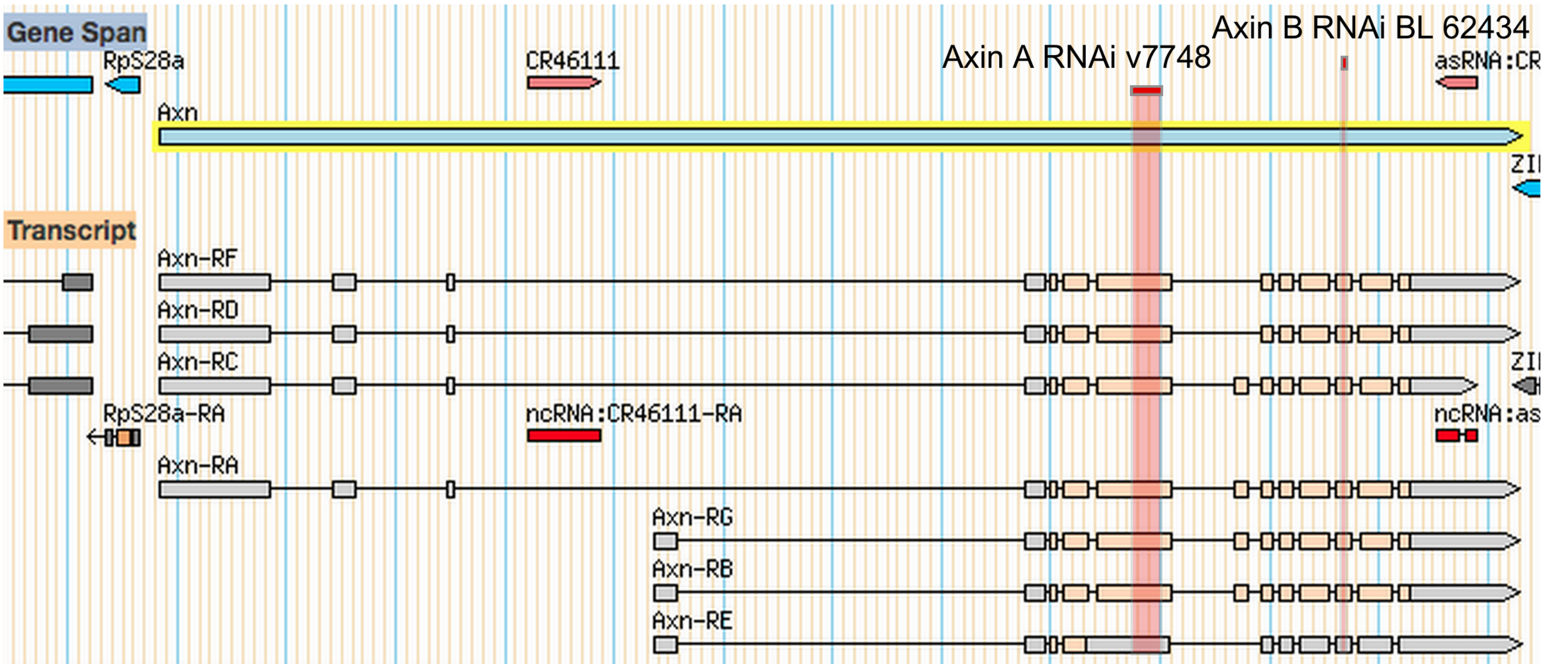

Axin B RNAi BL 62434
Axin A RNAi v7748
Gene Span
RpS28a
CR46111
asRNA:CR
Axn
Transcript
Axn-RF
Axn-RD
Axn-RC
RpS28a-RA
ncRNA:CR46111-RA
ncRNA:as
Axn-RA
Axn-RG
Axn-RB
Axn-RE

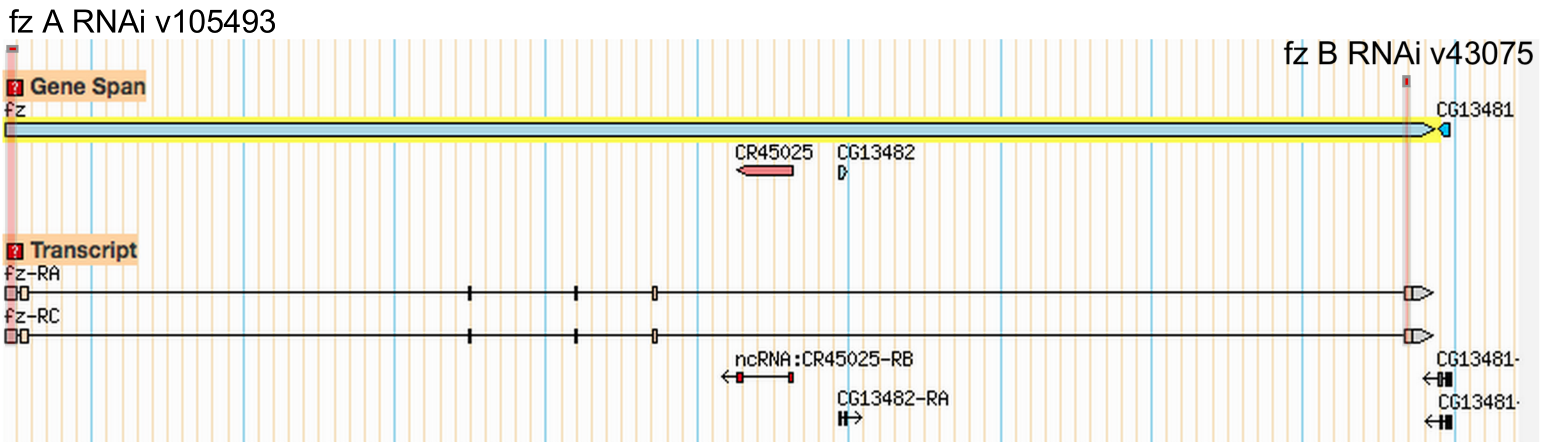

fz A RNAi v105493
fz B RNAi v43075
Gene Span
fz
CG13481
CR45025
CG13482
Transcript
fz-RA
fz-RC
ncRNA:CR45025-RB
CG13482-RA

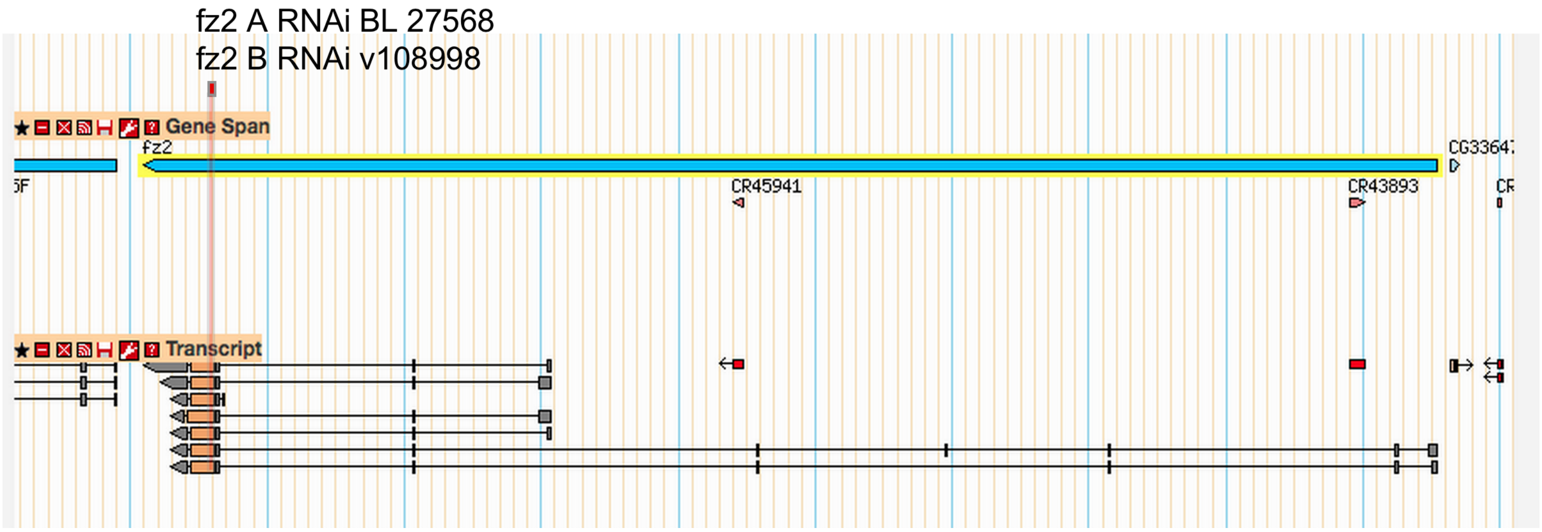

fz2 A RNAi BL 27568
fz2 B RNAi v108998
Gene Span
fz2
CR45941
CR43893
Transcript

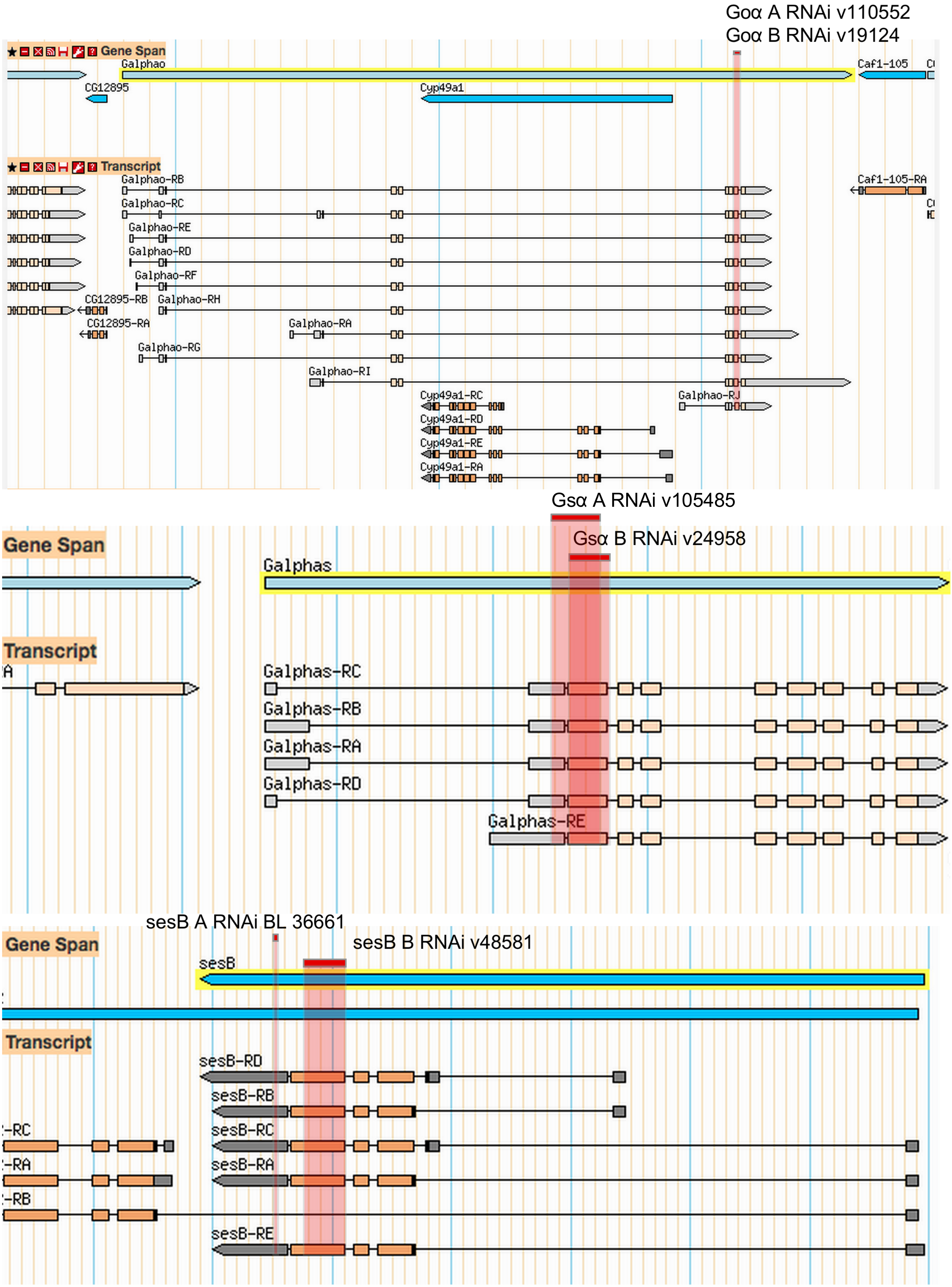
Goα A RNAi v110552
Goα B RNAi v19124
Gene Span
Galphao
CG12895
Cyp49a1
Caf1-105
Transcript
Galphao-RB
Galphao-RC
Galphao-RE
Galphao-RD
Galphao-RF
CG12895-RB
Galphao-RH
CG12895-RA
Galphao-RA
Galphao-RG
Galphao-RI
Cyp49a1-RC
Galphao-RJ
Cyp49a1-RD
Cyp49a1-RE
Cyp49a1-RA
Caf1-105-RA
Gsα A RNAi v105485
Gsα B RNAi v24958
Gene Span
Galphas
Transcript
Galphas-RC
Galphas-RB
Galphas-RA
Galphas-RD
Galphas-RE
sesB A RNAi BL 36661
sesB B RNAi v48581
Gene Span
sesB
Transcript
sesB-RD
sesB-RB
sesB-RC
sesB-RA
sesB-RE
